# Measuring Obesogenicity and Assessing Its Impact on Child Obesity: A Cross-Sectional Ecological Study for England Neighbourhoods

**DOI:** 10.3390/ijerph191710865

**Published:** 2022-08-31

**Authors:** Peter Congdon

**Affiliations:** School of Geography, Queen Mary University of London, Mile End Rd., London E1 4NS, UK; p.congdon@qmul.ac.uk

**Keywords:** obesogenic environments, obesity, spatial, latent construct, fast food, healthy food, access to parks and recreation

## Abstract

Both major influences on changing obesity levels (diet and physical activity) may be mediated by the environment, with environments that promote higher weight being denoted obesogenic. However, while many conceptual descriptions and definitions of obesogenic environments are available, relatively few attempts have been made to quantify obesogenic environments (obesogenicity). The current study is an ecological study (using area units as observations) which has as its main objective to propose a methodology for obtaining a numeric index of obesogenic neighbourhoods, and assess this methodology in an application to a major national dataset. One challenge in such a task is that obesogenicity is a latent aspect, proxied by observed environment features, such as poor access to healthy food and recreation, as well as socio-demographic neighbourhood characteristics. Another is that obesogenicity is potentially spatially clustered, and this feature should be included in the methodology. Two alternative forms of measurement model (i.e., models representing a latent quantity using observed indicators) are considered in developing the obesogenic environment index, and under both approaches we find that both food and activity indicators are pertinent to measuring obesogenic environments (though with varying relevance), and that obesogenic environments are spatially clustered. We then consider the role of the obesogenic environment index in explaining obesity and overweight rates for children at ages 10–11 in English neighbourhoods, along with area deprivation, population ethnicity, crime levels, and a measure of urban–rural status. We find the index of obesogenic environments to have a significant effect in elevating rates of child obesity and overweight. As a major conclusion, we establish that obesogenic environments can be measured using appropriate methods, and that they play a part in explaining variations in child weight indicators; in short, area context is relevant.

## 1. Introduction

Increases in child obesity, like those in adult obesity, have been linked both to changing dietary patterns and to reduced physical activity [1,2,3]. Changes in diet and activity are to some degree linked to immediate home and family environments [4]. However, broader influences in the neighbourhood environment, often called contextual influences, have been proposed as a major influence also, with environments that promote higher weight being denoted obesogenic [5,6]. Income and ethnic group differences are also important influences on obesity, potentially operating via neighbourhood factors [7,8,9]. For example, Friends of the Earth [10] find a strong correlation between green space deprivation and ethnicity in England, while an official US review [11] found that “certain racial and ethnic groups and low-income individuals and families live, learn, work, and play in places that lack health-promoting resources such as parks, recreational facilities, high-quality grocery stores, and walkable streets”.

Obesogenicity is a composite of several facets: thus, an official UK report [12] specifies the broad scope of obesogenic environments, namely “the term obesogenic environment refers to the role environmental factors may play in determining both nutrition and physical activity”. In that regard, factors such as the food environment, access to recreational green space and exercise opportunities, and settlement configuration (e.g., urban sprawl, walkability) have been suggested in meta-analytic reviews [13,14,15]. Findings on impacts of environmental attributes on obesity have been mixed, including weak or null effects. For example, the meta-review by Jia et al. [15] report a majority of relevant studies as suggest a positive association between fast food access and weight-related outcomes, but that meta-analysis does not demonstrate significant results. A review of the evidence regarding greenspace and obesity [16] found mixed or weak evidence of a relationship.

Many conceptual descriptions of obesogenic environments are available, but relatively few attempts have been made to quantify obesogenic environments (obesogenicity); see [17]. If one does focus on how to quantify obesogenicity, a methodological challenge is that obesogenicity is a latent quantity proxied by actually measured indicators, and that such indicators may play different roles in defining the underlying latent index, some reflecting it, others more appropriately seen as causal influences on it. In the analysis below we provide a novel approach to the analysis of obesogenic environments that reflects the different conceptual roles of observed indicators.

Furthermore, different indicators may have varying relevance in defining obesogenicity, and hence some way of assigning loadings to each indicator should be part of any methodology. Furthermore, like obesity itself, obesogenicity is expected to be spatially clustered as conceptual accounts stress certain neighbourhood types (e.g., low income areas) as having worse access to healthy food and recreation opportunities, and such neighbourhoods tend to be spatially clustered [18]. Obesity itself is spatially clustered [19], and one would expect obesogenic environments to be spatially clustered also.

The current study is an ecological study (using area units as observations), which has as its main objective to set out a method to measure obesogenic environments and to demonstrate its use on a major nationwide dataset, namely child overweight and obesity in neighbourhoods (small areas) across England. Establishing that obesogenicity is spatially clustered (and incorporating the potential for this into the proposed method) is a subsidiary objective. Our approach uses two stages: first, a measurement model is used to develop a numeric index of obesogenic environments from observed neighbourhood indicators. The second stage is a regression of obesity and overweight rates on the numeric index of obesogenicity and other relevant variables. Two types of measurement model are used, one using continuous observed indicators, the other uses binary indicators of neighbourhood subset type.

The papers by Kaczynski et al. [17] and Wende et al. [20] develop obesogenicity indices using percentiles on each of multiple indicators, and then summing the percentiles to obtain a summary score. The approach in these papers assigns equal weight to all the indicators used in constructing a score for obesogenic environments, whereas in fact some may be more relevant than others. It also does not incorporate potential spatial clustering in obesogenic environments.

Child obesity, and trends in it, have major political salience in England. Thus a summary [21] of recent changes in these child measures, collected under the National Child Measurement Programme (NCMP), mentions that “unprecedented increases were seen in the prevalence of obesity”. The child measures are obtained for all children in state maintained primary schools (i.e., there is no element of sampling involved); around 94% of primary school pupils in England are in state schools [22]. The area framework is provided by 6791 neighbourhoods, called Middle Level Super Output Areas (MSOAs), which provide a complete coverage of England [23] Section 3.

We find the obesogenicity index to have a significant non-negligible effect on obesity and overweight in English neighbourhoods, secondary in importance to impacts of income deprivation, but partly mediating the deprivation effect. We find different observed indicators to have differing relevance in defining obesogenicity, and also that obesogenicity is spatially clustered.

Our goal in the analysis can be summarized as seeking to encapsulate aspects of the obesogenic environment in a summary numerical score, obtained using appropriate methods. Many existing studies use a single regression analysis with obesity on the one hand and various indices measuring selected obesogenic features as predictors on the other. Among other limitations (e.g., collinearity between different aspects of obesogenic environments), these studies may not capture the full role of social stratification in structuring obesogenicity and hence obesity.

The layout of the rest of the paper is as follows. Section 2 sets out the conceptual distinction between different types of indicator, without formally describing methods. This is the basis for a schematic representation of the model elements. Section 3 describes the case study; Section 4 sets out methods and rationale for choice of variables; Section 5 sets out the results; and a final Discussion section contains conclusions from the research and possible limitations on the study.

## 2. Measuring Obesogenic Environments: Formative and Reflexive Indicators

As mentioned above, obesogenicity is a latent quantity proxied by actually measured indicators, and such indicators may play different roles in defining the underlying latent index, some reflecting it, others more appropriately seen as causal influences on it. A relevant distinction in the analysis of latent constructs is that between reflexive and formative indicators [24,25]. Formal quantitative methods allowing latent constructs defined by both reflexive and formative indicators have been applied in many settings, for example in marketing [26], albeit not (so far as the authors are aware) in defining obesogenicity.

Thus reflexive indicators of obesogenicity are neighbourhood indicators taken on the basis of accumulated research to either increase (e.g., fast food access), or diminish (e.g., recreation and green space access), as obesogenicity increases. By contrast, formative indicators include socio-structural aspects more typically associated with obesogenic environments, and possibly to some degree causal influences on them. Different social and demographic groups have unequal access to healthy environments [26], a phenomenon denoted as environmental injustice. Hence, social stratification is likely to define the spatial environment for obesity, and it is important that its role is included in any method to summarise obesogenicity.

As an illustration of this distinction, the Congressional Research Service (CRS) [27] in the US uses a subset identification approach incorporating the income effect on obesogenic environments. It uses the joint occurrence of low income (which can be seen as a formative indicator), and low access to healthy food (a reflexive indicator) in US census tracts to define food deserts.

**Figure 1:**
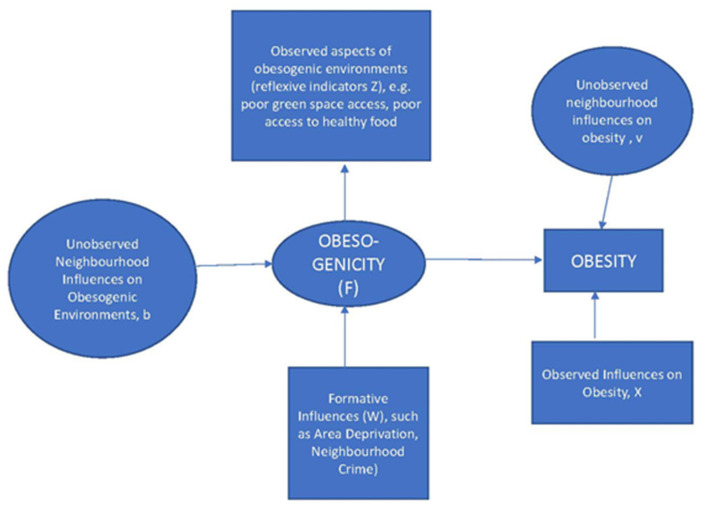
Diagrammatic Representation of Postulated Influences on Obesity.

Obesogenic environments are only one potential factor in explaining neighbourhood variations in overweight and obesity. Impacts of obesogenic environments on these outcomes may be moderated by other neighbourhood characteristics, such as area deprivation and aspects of the social environment [28]. Some of these may also figure as formative influences on obesogenic environments. In addition, unmeasured aspects of neighbourhoods may influence both obesogenicity and obesity. Figure 1 accordingly shows the postulated interrelationships in the present study between observed indicators (rectangles) and latent variables.

## 3. Case Study and Study Design

In this paper we employ environmental indicators to develop a numeric index of obesogenicity, and assess impacts of obesogenicity on child obesity and overweight on children (ages 10–11) in English neighbourhoods. The spatial framework is provided by 6791 MSOAs, which provide a complete coverage of England and have an average population (of all ages) of 8200. Nested within MSOAs around 33,000 smaller are areas known as lower super output areas (LSOAs).

The obesity and overweight rates (percentages) are for 2019–2020, provided under the UK National Child Measurement Programme [21], and released without child population denominator information. As mentioned earlier, child weight measures are obtained for all children in state maintained primary schools.

Hence, there is no sampling element in either the neighbourhood environment indicators (which are for all England neighbourhoods, not a subsample), or the obesity rates (which are measured for all children in state schools, not a subsample).

The study design is observational and is a cross-sectional ecological study, using areas as the unit of analysis. An ecologic or aggregate study focuses on the comparison of groups (such as areas), rather than individuals [29]. Following the terminology of Morgenstern [29] page 66, the design is an analytic multiple-group study, where “we assess the ecologic association between the average exposure level or prevalence and the rate of disease among many groups”. In terms of the classification of study designs presented by Song and Chung [30], the analysis we undertake here is a retrospective comparative observational study.

## 4. Methods 

### 4.1. Choice of Formative Indicators 

We now consider in detail the variables used in the case study and the rationale for their inclusion. Thus the role of formative influences in UK studies of obesity is apparent in that low area socio-economic status (i.e., high area deprivation) are associated with less healthy environments in general, including worse access to healthy food, recreation sites and parks, and exercise opportunities [31,32]. With regard to food access, Maguire et al. [33] in a longitudinal UK study report that “the most deprived wards [small areas] had the highest mean density of takeaway food outlets at every time point”. With regard to physical activity according to income and deprivation, a report by the Government Office for Science [12] states that “deprivation and poverty were found to be associated with low levels of leisure-time physical activity in a number of studies”.

The income effect on physical activity may be partly bound up with variations in neighbourhood safety (actual and perceived). Impacts of diminished safety on child overweight are reported in a number of studies [34,35,36], operating via restrictions on physical activity levels. Perceived safety is likely to reflect crime levels, and neighbourhood crime has also been found to impact on child obesity [37,38].

Ethnicity is another sociodemographic variable related to obesogenicity. In the US, black ethnicity is associated with worse access to healthy food [39,40], while for the UK, the Active Lives Surveys show that percentages of adults eating five portions of fruit and vegetables daily to be lower among ethnic groups [41]. Worse access to recreation for ethnic minorities has been reported. Regarding child physical activity in particular, Sport England [42] finds that black children are less likely (35%) to be physically active than white British children (47%), reflecting worse access to outdoor exercise space.

There is also evidence that the presence or not of obesogenic environments is related to urban/rural status [43]. The study by Kaczynski et al. [17] in fact finds US rural areas to be more obesogenic, but evidence for the UK is lacking. One would expect an obvious impact of urban–rural status on access to both fast food outlets and supermarkets, with worse access in rural areas, regardless of area deprivation. This is simply because more rural areas are more distant from a range of services, including all types of food outlet. This potentially distorting effect should ideally be corrected for in deriving a summary index of obesogenicity.

As formative indicators in the analysis here we use income deprivation, a clear measure of neighbourhood socio-economic status [44]; the proportion of children aged 10–14 in each MSOA who are white; and a measure of rurality based on a UK Census eight-fold category of urban–rural status [45].

### 4.2. Measuring Obesogenic Environments: Reflexive Indicators

A wide range of observable indicators have been suggested as reflexive of obesogenic environments. Regarding the role environmental factors play in determining nutrition, food deserts have been defined especially in terms of varying spatial access to healthy food outlets (such as supermarkets) as against less healthy outlets (e.g., fast food providers).

A UK study by Cetateanu and Jones [46] confirmed that greater access to unhealthy food outlets was associated with child overweight, and that more unhealthy food outlets were located in deprived areas. However, in regression analysis, this study found that unhealthy food outlets only slightly explained (i.e., mediated) the association between weight status and deprivation in older children. Some studies [33,47] in fact report that supermarket access is not necessarily worse in deprived areas, at least in the UK.

Access to recreation opportunities have also been found relevant to explaining variations in child obesity [48,49]. In particular, better access to private garden space has been linked to lower child obesity [50]. The primary mechanism for the impact of recreation and greenspace access on overweight is through increased opportunities for physical activity [51,52].

Sprawl and walkability have also been implicated in explaining area overweight variations, especially in geographically extensive nations (e.g., the US, Australia, Canada). Urban sprawl is typified by low density suburban development with high automobile dependence and restricted walkability [53]. However, findings regarding walkability and obesity for the UK are mixed [43], may depend on definitions [54], may be at odds with other aspects of obesogenicity, and may be subject to anomalies in defining walkability for lower density and rural areas. The study by Burgoine et al. [43] reports that “despite strong correlations between residential density, street connectivity and land use mix, the latter two factors failed to exhibit an association with BMI”. The study by Stockton et al. [55] reports walkability in London as increasing towards the metropolitan centre, despite such areas being characterized by lower green space access, e.g., [10].

In practice four reflexive indicators of the food environment are used: fast food density in the local neighbourhood (MSOA) itself [56]; average fast food density in adjacent neighbourhoods; proximity (inverse distance) to nearest fast food outlet [57]; and travel times to supermarkets or general food stores, from the 2019 Index of Multiple Deprivation [44]. It would be expected that all these indicators would increase as obesogenicity does. As indicators of recreation/park access, the three indices used are: garden area per capita and total green space per capita, both based on data from the FOE study [10]; and an active green space access index, as defined in the Access to Healthy Assets and Hazards (AHAH) dataset [57]. This is based on the distance to the nearest greenspace conducive to physical activity, including public parks or gardens, play spaces, playing fields, and tennis courts.

### 4.3. Form of Analysis: First Measurement Model

In the study here, we model the derivation of the obesogenic environment score in a separate first stage, an obesogenicity measurement model. Two types of measurement model are considered, as discussed in this and the next section. We then model the impact of the environment score (from each type of measurement model) on indicators of overweight and obesity in a separate stage (see Section 4.5).

We consider two forms of measurement model. In the first model to measure the latent obesogenic environment, reflexive indicators, denoted Z_ij_ (for areas i and j = 1, …, J indicators) are all continuous and assumed to be normally distributed, with loadings λ_j_ of indicator j on the common (obesogenicity) factor score F_i_ for the i-th MSOA. The J = 7 indicators, relating to food and recreation environments, are as described in Section 4.2.

The F scores are also taken to be normally distributed and to depend on K = 4 formative indicators: income deprivation provided by the Ministry of Housing, Communities and Local Government (MHCLG) [44]; the proportion of 10–14 year olds who are white; a measure of neighbourhood crime [44]; and a measure of rurality, namely the ridit score [58], based on an eightfold ordinal urban–rural categorisation of MSOAs [45]. The formative indicators W_ik_ (for K indicators) have coefficients δ_k_ in the formative model.

The F scores also depend on a random spatial term, b_i_ defined as in Langford et al. [59]. In this way both observed area characteristics, and unobserved (spatially clustered) influences on obesogenicity are included in the definition of the scores on the latent construct. We would expect neighbourhoods geographically close to each other to have similar levels of obesogenicity.

The mathematical form of such models (albeit excluding spatial clustering effects) is discussed in the studies by Bollen and Diamantopoulos [24] and Ghosh and Dunson [60]. In such models the impact of formative indicators is via a type of regression (with coefficients δ_k_). The mathematical form in the approach used here, as the first measurement model, can be summarised as

Z_ij_ = α_j_ + λ_j_F_i_ + e_ij_            j = 1, …, J

F_i_ = W_i1_δ_1_ + …+ W_ik_δ_k_ + b_i_ + u_i_

where e_ij_ and u_i_ are normally and independent and identically (iid) distributed with zero means. The W_ik_ are standardised, so that the δ_k_ coefficients can be compared to show the relative importance of the formative factors. The Z_ij_ are also standardized so the loadings λ_j_ show how important each reflexive indicator is in defining obesogenicity. The value of the spatial effect b in neighbourhood i is obtained as a weighted average of values in adjacent areas, i.e.,

b_i_ = _j_w_ij_b_j_*/ _j_w_ij_

where the b* are iid normal, and the w_ij_ are spatial interactions (with w_ij_ = 1 for adjacent neighbourhoods, w_ij_ = 0 otherwise). All loadings are taken as unknown, with λ_1_ constrained to be positive to ensure consistent identification, and the variance of b* and u is therefore set to 1.

### 4.4. An Alternative Measurement Model: Representing Subsets of Neighbourhoods

The approach to a measurement model in Section 4.3 can be seen as a conventional one, albeit distinguishing between reflexive and causal indicators. We investigate here an alternative approach, reflecting that average associations do not necessarily fit paradigmatic representations of obesogenic environments, and a neighbourhood subset approach may be better adapted to identify exceptions to the average pattern. Thus while the overall relationship between supermarket access and area deprivation in English neighbourhoods does not necessarily fit the paradigm representations [33,47], there may still be a subset of deprived areas in England with poor supermarket access.

To identify such areas, and similarly obesogenic environments defined by other characteristics, we adopt and extend the subset identification approach adopted to identify food deserts in the US [27]. Specifically, we define binary measures according to either (a) poor supermarket access (or fast food proximity) or (b) poor recreation/park access, coupled with either (c) high area deprivation, or (d) high non-white percentages among children aged 10–14. That is we define binary measures which include reflexive and formative aspects. For parsimony, we focus especially on area income deprivation and ethnicity as formative indicators relevant to defining the binary measures, these being most relevant in assessing the role of social stratification in obesogenicity.

We also define an indicator for low density, lower income, suburban areas with car commuting reliance to represent urban sprawl. Car commuting data are from the UK Census.

So nine binary indicators are defined according to whether a neighbourhood has:

1. Above average area income deprivation and above average distance to supermarket or food store
2. Above average area income deprivation and above average fast food proximity
3. Above average non-white percentages and above average distance to supermarket or food store
4. Above average non-white percentages and above average fast food proximity
5. Above average area income deprivation and below average access to private green space
6. Above average area income deprivation and below average access to active green space
7. Above average non-white percentages and below average access to private green space
8. Above average non-white percentages and below average access to active green space.
9. Above average income deprivation and car commuting, but below average population density (for metropolitan and other urban MSOAs only).

In the definitions above, food access, green access, commuting and density are defined relative to averages for grouped urban–rural category, or RUC11, for short [45], namely: metropolitan neighbourhoods (RUC11 categories 1 or 2); other highly urban (RUC11 3 or 4); rural fringe (RUC11 5 or 6); and sparsely settled rural (RUC11 7 or 8). In this way the varying access to services effect linked to urban–rural category is controlled for. For example, one may with this approach more readily identify rural areas with relatively obesogenic features, as compared to other rural areas.

The binary measures above are now the observed or manifest indicators used to define the obesogenic area construct [61]. By virtue of the way the indicators are defined, information on both reflexive and formative indicators is retained, but used in a more goal oriented way. So this model no longer involves formative regression of the construct on income deprivation and white ethnicity, since information on these is incorporated in the indicators. However, the construct scores are still centred around a spatially correlated random effect to represent the effect of unmeasured spatially clustered influences on obesogenic environments, as defined in [59].

Mathematically the second measurement model is represented (for J = 9) as

Z_ij_~ Bernoulli(η_ij_)         j = 1, …, J

logit(η_ij_) = α_j_ + λ_j_F_i_

F_i_ = b_i_ + u_i_

where the Z_ij_ are binary, and b_i_ and u_i_ have the same specification as discussed above. As previously, all loadings are taken as unknowns, with λ_1_ constrained to be positive to ensure consistent identification, and the variance of b* and u set to 1.

### 4.5. Regression of Obesity and Overweight on Obesogenicity Scores and Other Area Risk Factors 

In the second stage regression, we use the estimated obesogenicity scores F_i_ (from each of the two measurement models), and regress the log odds of obesity or all overweight on these scores, and also on income deprivation, white ethnicity (ages 10–14), neighbourhood crime, and rurality. Impacts of deprivation on child obesity are widely reported [8,47], as is relatively higher obesity among non-white children [9]. Neighbourhood crime has also been found to impact on child obesity [37,38]. Urban–rural status has also been found to impact child obesity, e.g., [62]. Note that these four predictors are also used as reflexive indicators in the first measurement model.

The predictors of obesity are converted to standardized form so that their relative importance can be established. The impact of these area risk factors, collectively denoted X in Figure 1, is therefore expressed via standardized regression coefficients (β parameters), and by the relative risks of obesity (or overweight) when comparing high values (95th percentile) vs. low values (5th percentile) of each risk factor. A spatial error term v, representing unobserved spatially correlated influences on obesity or overweight, is also included in the regressions for obesity and overweight, as per the scheme in [59].

To measure the goodness of fit of the regression we use the Widely Applicable Information Criterion, WAIC [63], which is lower for better fitting models. Estimation uses Bayesian techniques, as in the BUGS program [64]. Inferences are based on the second half of two chain runs of 10,000 iterations, with convergence assessed using Brooks-Gelman-Rubin criteria [65]. Regression coefficients and loadings are assigned *N*(0, 10) priors, apart from the first loading, assigned an exponential prior with mean 1. Precisions (inverse variances) are assigned gamma priors with shape 1 and rate 0.01.

## 5. Results

### 5.1. First Measurement Model: Loadings and Formative Regression Results

Table 1 shows the λ reflexive loadings, and δ formative coefficients, of the first measurement model. It can be seen that access to fast food is associated with increasing obesogenic environment scores (λ_1_, λ_2_ and λ_3_ are all positive). The highest loading among the food access indicators is for the fast food proximity index. By contrast, higher recreation/park access is negatively associated with obesogenicity, as would be expected. The highest negative loading on the recreation/park indicators is for access to private green space, including gardens.

Regarding impacts of formative indicators, it can be seen that income deprivation in areas is associated with higher obesogenicity scores, but this effect (a δ coefficient of 0.10) is relatively small compared to that of neighbourhood crime (0.70) in boosting obesogenicity. This suggests that crime (higher in deprived areas) mediates much of the direct deprivation effect on obesogenic environments. Impacts of higher levels of white ethnicity among children are a significantly negative influence on obesogenic environments, as are high levels of rurality. Alternatively stated, urban areas with high proportions of children in non-white ethnic groups are much more likely than average to be obesogenic.

The exception to the pattern of loadings and formative impacts in expected directions in Table 1 (according to obesity paradigm representations) is the negative loading on distance to a supermarket or food store. This reflects the fact that while fast food access is higher in deprived English neighbourhoods, access to a supermarket or food store is not worse in such neighbourhoods (in terms of overall association and correlation). On the latter feature, see studies such as those by Maguire et al. [33] and Smith et al. [47]. In fact, average distances to supermarkets are shorter in the 10% most deprived areas than in the 10% least deprived areas.

The results in Table 1 confirm, as discussed in the Introduction, that different indicators have varying relevance in defining obesogenic environments. Furthermore, confirmed is spatial clustering in such environments. The Moran spatial correlation index [66] for the obesogenicity scores from the first measurement model is 0.81, and a significance test under randomization, using the procedure moran.test in R gives a *p*-value of *p*-value under 2.2 × 10^−16^.

### 5.2. Alternative Measurement Model: Results

Table 2 shows the results of the measurement model when obesogenicity is measured using the nine binary indicators. It shows there are positive loadings for all observed binary indicators on the underlying construct. So all sub-categories of areas represented by the binary indicators represent various aspects of a single obesogenic environment construct, albeit to varying extents. The indicator for sprawl has a relatively low loading, suggesting that any sprawl effect is comparatively low for England, as compared to counties such as the US [67].

The high loadings involving non-white ethnicity show the centrality of ethnicity, as well as income deprivation, in defining obesogenic environments. Overall the role of social stratification (associated with income and ethnicity) in defining the spatial framework for obesogenicity is confirmed.

The high loadings for non-white ethnicity combined with distance to supermarkets and active recreation/parks reflect ethnic gradients in these environmental characteristics (higher supermarket distances and worse active green space access in areas with more children in non-white groups).

Again it is confirmed that different indicators have varying relevance in defining obesogenic environments. As to spatial clustering, the Moran spatial correlation index for the obesogenic environment index obtained using this approach is 0.49, with a *p*-value again under 2.2 × 10^−16^.

**Figure 2:**
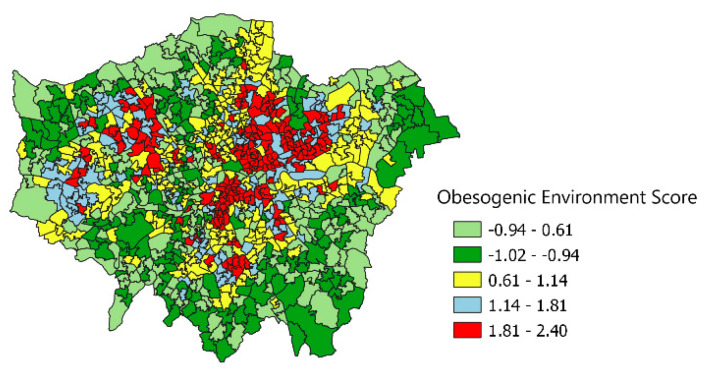
Greater London. Obesogenic Environment Index. Second Measurement Model.

To depict how this appears in visual terms, we map out the obesogenicity index in one English region, namely Greater London (see Figure 2). Higher values of the score are seen to cluster in inner east and south London especially. Lower scores occur throughout London but are most apparent in suburban areas, again with clustering. The Moran coefficient for spatial clustering of obesogenicity within London is 0.61, and is again highly significant.

### 5.3. Obesity and Overweight in Relation to Obesogenicity

As mentioned above, we use the obesogenic environment scores together with other relevant predictors, in a regression analysis of obesity and overweight rates from the NCMP. We convert the original percentage rates of obesity and overweight from the NCMP to log-odds and assume in the regression stage that the log-odds are normally distributed [68].

Table 3 shows the regression effects on obesity and overweight among children aged 10–11 of obesogenicity scores from the first measurement model, together with other area characteristics (X variables).

Table 3 shows the effect of income deprivation is paramount, with the standardized coefficient three times that for the obesogenic environment score, though both coefficients are significant in the sense that their 95% intervals are positive. The deprivation effect is stronger on obesity than on all overweight. The relative risks of obesity (comparing areas with high deprivation and low deprivation) are 68% higher in highly deprived areas. For all overweight this excess risk falls to 40%.

Whereas crime levels are a major influence on obesogenicity, they are a lesser influence (though still a significant area risk factor) for obesity and overweight as compared to deprivation per se.

Higher levels of white ethnicity among children are associated with lower obesity and overweight, but the ethnicity effect is relatively small as compared to that of deprivation. Effects of rural location on obesity and overweight are not significant.

Results from the regression stage based on the obesogenicity score from the second measurement model are shown in Table 4. It can be seen that income deprivation remains the paramount influence. However, the β coefficient for obesogenicity is higher than in Table 3, and also higher when compared to that for income deprivation. The standardized coefficient on obesogenicity in terms of impact on all overweight is now about half that for income deprivation. As compared to Table 3, the impact of income deprivation on child obesity and overweight is somewhat attenuated.

It can be seen from a reduced regression model (results not shown in detail) that some of the income deprivation effect is mediated by obesogenicity. If the obesogenicity score is omitted from the obesity regression, the standardized coefficient on income deprivation is raised from 0.168 to 0.222, and the relative risk for deprivation rises from 1.53 to 1.74. The change in standardized coefficients suggests that around 25% of the impact on child obesity of income deprivation is mediated by obesogenic environments.

It can also be seen that the fit (as measured by the WAIC in Table 4 as against Table 3) is improved when this way of measuring obesogenic environments is used. One aspect of the better fit is that the correlation between the obesogenic environment score and child obesity is 0.65 using the scores from the second measurement model, as compared to 0.54 using the scores from the first measurement model.

### 5.4. Results: Obesogenicity Profiles

**Figure 3:**
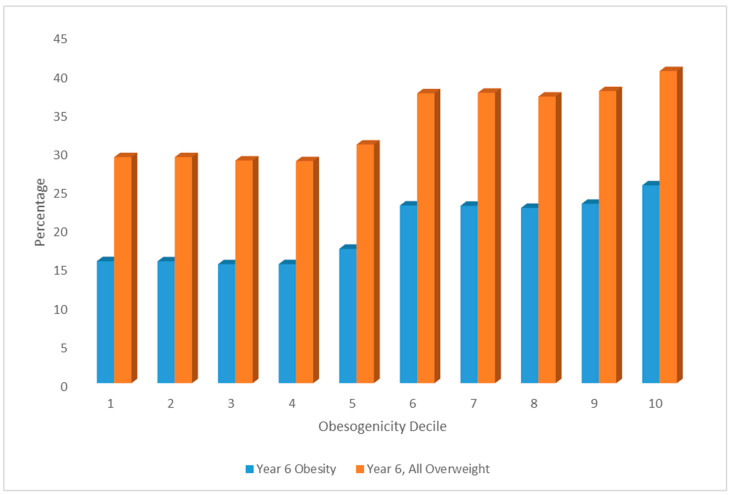
Child Obesity and Overweight according to Obesogenicity Decile.

Figure 3 shows gradients in child obesity and overweight for deciles of the obesogenicity score (under the better fitting second measurement model). The average child obesity rate in the most obesogenic neighbourhoods is 25.5%, as compared to 15.7% obesity in the least obesogenic.

As one aspect of the spatial patterning of obesogenicity, it can be seen from Table 5 that the second method for measuring obesogenic environments includes some relatively rural and suburban fringe areas as obesogenic, though highly urban areas are the most likely to be obesogenic. About two thirds of the most urban areas (the first two categories of the eight in Table 5) have obesogenic scores above the median.

## 6. Discussion and Conclusions

Many studies of obesity, both among adults and children, have focused on particular facets of obesogenic environments, such as fast food outlets, or active green space. The present study has instead sought to consider the impact of the obesogenicity in an inclusive and comprehensive sense, following definitions such as that proposed by the Government Office for Science [12], namely ”the role environmental factors may play in determining both nutrition and physical activity”.

There are relatively few studies which have attempted to define a comprehensive obesogenic environment index, exceptions being studies such as those of Kaczynski et al. [17] and Wende et al. [20], and the present study has considered this measurement question as a priority. The present study is distinctive in using multivariate methods to obtain an obesogenic environment score, while also recognizing (a) that some indicators are more important than others in defining such environments, and (b) that obesogenicity is potentially spatially clustered. Appropriate methods, as proposed in the study here, should accommodate these features.

The present study has also argued that any definition or measurement of obesogenic environments should reflect their association with particular “formative” socio-demographic contexts: obesogenic environments tend to be associated with deprived neighbourhoods, and (in the UK and US) with neighbourhoods having concentrations of ethnic minority groups. This facet of obesogenic environments feeds into some definitions of such environments, for example, the official definition of food deserts in the US by the Congressional Research Service [27].

The methodological implication of these two considerations (i.e., inclusivity and formative-contextual relevance) has been taken forward in two alternative approaches to measuring obesogenicity in the study here. One has been by a full model with both reflexive and formative indicators, following the conventional mathematical approach [24,25]. The other has been a novel one, namely to define multiple binary indicators which reflect particular aspects of an obesogenic environment (cf. [27]), regarding nutrition and physical activity access on the one hand, and formative-contextual factors on the other (e.g., deprived area or not). The latter approach can be extended flexibly beyond the particular set of indicator definitions used in the present paper. For example, one could define an indicator for neighbourhoods which have both above average fast food access, below average recreation/park access, and above average deprivation.

When the resulting obesogenic environment scores have been combined with income deprivation in regression models seeking to explain child obesity or overweight, it has been found that obesogenicity retains a significant, albeit secondary, effect. The standardized coefficients for the obesogenic environment score (under the binary indicators approach) are around a half of the coefficients on income deprivation. Reduced regression indicates that some of the effect of deprivation on obesity is mediated by obesogenicity.

The broader implication of the present study is the need to consider suitable multivariate methods to measure the latent construct of obesogenicity as a neighbourhood characteristic. Any index may depend to some degree on the country being studied, the indicators being used, and the measurement method. However, certain principles are implied by the present study: such as the relevance of the formative context, typically aspects of societal stratification [28], as well as reflexive indicators of food and activity access.

The present study has the limitation that it is an ecological and observational study so any findings about impacts of say, fast food access, or private green space access, cannot be taken as relevant to individual level causation of obesity. It is a cross-sectional study whereas stronger inferences may be obtained by a longitudinal, albeit still observational, analysis. Furthermore, the findings from the present study are conditional on the indicators used, namely readily (and freely) available indicators at a particular spatial scale for English neighbourhoods.

Regardless of the limitations of the set of indicators used, those available have been used to provide and illustrate a feasible measurement approach to obesogenic environments, one which shows that such environments significantly affect child obesity and overweight. Thus despite some skeptical assessments [69] regarding environmental impacts on obesity, the study here adds to the weight of evidence that context matters.

## Figures and Tables

**Table 1:** First Measurement Model for Obesogenicity.

Coefficient Estimates and 95% Intervals			
*Reflexive Model, Loadings on:*	Estimate	2.5%	97.5%
Density Fast Food Outlets, Local Area	0.217	0.206	0.228
Density Fast Food Outlets, Surrounding Area	0.240	0.229	0.251
Proximity to Fast Food Outlets	0.381	0.371	0.392
Distance to Supermarkets, General Food Stores	−0.337	−0.348	−0.327
Access to Private Greenspace	−0.459	0.450	0.469
General Green Space Access	−0.286	0.275	0.297
Active Green Space Access	−0.276	0.265	0.287
*Formative Model, Standardized Regression Coefficients*	Estimate	2.5%	97.5%
Income Deprivation	0.101	0.055	0.148
White ethnicity, Children 10–14	−0.689	−0.733	−0.647
Crime Index	0.701	0.649	0.754
Rurality	−0.688	−0.732	−0.643

**Table 2:** Obesogenicity, Second Measurement Model, Loadings on Binary Indicators.

*Binary Indicator (=*1 *if Area Satisfies Criterion,* 0 *Otherwise)*	Estimate	2.5%	97.5%
Above average area income deprivation & above average distance to supermarket/food store	1.46	1.37	1.55
Above average area income deprivation & above average fast food proximity	1.62	1.50	1.74
Above average non-white percentages & above average distance to supermarket or food store	5.14	4.61	5.73
Above average non-white percentages & above average fast food proximity	3.72	3.39	4.10
Above average area income deprivation & below average access to private green space	1.17	1.09	1.26
Above average area income deprivation & below average access to active green space	1.55	1.45	1.66
Above average non-white percentages & below average access to private green space	2.54	2.34	2.75
Above average non-white percentages & below average access to active green space	6.66	5.75	7.71
Above average income deprivation & car commuting, but below average population density (metropolitan & other urban areas only)	0.44	0.37	0.51

**Table 3:** Child Obesity and Overweight Regressions. Obesogenicity First Measurement Model.

Obesogenic Environment Score from First Measurement Model			
*(A) Obesity*			
Impacts of area risk factors (β coefficients)			
	Estimate	2.5%	97.5%
Obesogenic Environment Score	0.069	0.062	0.076
Income Deprivation	0.207	0.192	0.224
White Ethnicity (Children 10–14)	−0.033	−0.046	−0.020
Rurality Index	0.008	−0.003	0.018
Crime Deprivation	0.039	0.025	0.054
Impacts of area risk factors (as Relative Risks)			
	Estimate	2.5%	97.5%
Obesogenic Environment Score	1.21	1.19	1.24
Income Deprivation	1.68	1.62	1.75
White Ethnicity (Children 10–14)	0.92	0.89	0.95
Rurality Index	1.02	0.99	1.04
Crime Deprivation	1.11	1.07	1.15
Fit measure			
WAIC	6344		
*(B) All Overweight including Obesity*			
Impacts of area risk factors (β coefficients)			
	Estimate	2.5%	97.5%
Obesogenic Environment Score	0.056	0.050	0.064
Income Deprivation	0.165	0.154	0.175
White Ethnicity (Children 10–14)	−0.030	−0.039	−0.023
Rurality Index	0.007	−0.002	0.015
Crime Deprivation	0.025	0.016	0.034
Impacts of area risk factors (as Relative Risks)			
	Estimate	2.5%	97.5%
Obesogenic Environment Score	1.14	1.12	1.16
Income Deprivation	1.40	1.37	1.43
White Ethnicity (Children 10–14)	0.94	0.92	0.95
Rurality Index	1.01	1.00	1.03
Crime Deprivation	1.06	1.03	1.08
Fit measure			
WAIC	7617		

**Table 4:** Obesity and Overweight Regressions. Obesogenicity Second Measurement Model.

Obesogenic Environment Score, Second Measurement Model			
*(A) Obesity*			
Impacts of area risk factors (β coefficients)			
	Estimate	2.5%	97.5%
Obesogenic Environment Score	0.071	0.064	0.079
Income Deprivation	0.168	0.156	0.180
White Ethnicity (Children 10–14)	−0.028	−0.040	−0.012
Rurality Index	−0.024	−0.035	−0.013
Crime Deprivation	0.047	0.034	0.063
Impacts of area risk factors (as Relative Risks)			
	Estimate	2.5%	97.5%
Obesogenic Environment Score	1.18	1.15	1.20
Income Deprivation	1.53	1.48	1.57
White Ethnicity (Children 10–14)	0.93	0.90	0.97
Rurality Index	0.95	0.92	0.97
Crime Deprivation	1.13	1.10	1.18
Fit measure			
WAIC	6258		
*(B) All Overweight including Obesity*			
Impacts of area risk factors (β coefficients)			
	Estimate	2.5%	97.5%
Obesogenic Environment Score	0.065	0.054	0.075
Income Deprivation	0.130	0.117	0.144
White Ethnicity (Children 10–14)	−0.023	−0.032	−0.014
Rurality Index	−0.021	−0.030	−0.012
Crime Deprivation	0.030	0.018	0.040
Impacts of area risk factors (as Relative Risks)			
	Estimate	2.5%	97.5%
Obesogenic Environment Score	1.13	1.11	1.15
Income Deprivation	1.31	1.27	1.35
White Ethnicity (Children 10–14)	0.95	0.93	0.97
Rurality Index	0.96	0.94	0.98
Crime Deprivation	1.07	1.04	1.09
Fit measure			
WAIC	7590		

**Table 5:** Obesogenic Environment Score and Urban-Rural Context. Binary Indicators Measurement Model.

Neighbourhood Category (RUC11)	Quartile 1 (Low Obesogenicity)	Quartile 2	Quartile 3	Quartile 4 (High Obesogenicity)	Total Neighbourhoods in Category	% Above Median Obesogenicity
Urban: Major Conurbation	374	425	772	828	2399	67
Urban: Minor Conurbation	42	35	147	25	249	69
Urban: City & Town	786	720	628	804	2938	49
Urban: City & Town, Sparse Setting	1	4	6	2	13	62
Rural Town & Fringe	219	232	113	24	588	23
Rural Town & Fringe, Sparse Setting	4	6	9	1	20	50
Rural Village & Dispersed	278	231	28	2	539	6
Rural Village/Dispersed, Sparse Sett’g	18	20	7	0	45	16

## Data Availability

Data available on public sites of Office of National Statistics and ESRC Consumer Data Research Centre.
